# Adenosine Attenuates LPS-Induced Cardiac Dysfunction by Inhibition of Mitochondrial Function via the ER Pathway

**DOI:** 10.1155/2019/1832025

**Published:** 2019-01-10

**Authors:** Mengnan Zeng, Beibei Zhang, Benke Li, Yuxuan Kan, Shengchao Wang, Weisheng Feng, Xiaoke Zheng

**Affiliations:** ^1^Henan University of Chinese Medicine, Zhengzhou 450046, China; ^2^Collaborative Innovation Center for Respiratory Disease Diagnosis and Treatment & Chinese Medicine Development of Henan Province, Zhengzhou 450046, China

## Abstract

Sepsis is a life-threatening organ dysfunction syndrome with a high rate of mortality. It is caused by an abnormal immune response to infection, and the occurrence of sepsis-induced cardiomyopathy is the primary cause of death. The present study was designed to examine the effects of adenosine on lipopolysaccharide- (LPS-) induced cardiac anomalies and the underlying mechanisms involved. Adenosine (25, 50, and 100 mg/kg, i.g., 2 times/day) was administered for three days, followed by the induction of sepsis by intraperitoneal injection of LPS (10 mg/kg/2h). The effects of adenosine on inflammatory factors, LVEF, LVFS, and MAPK in septic rats (half male and half female) were observed. Subsequently, the effect of adenosine (10 *μ*M) on the mitochondrial function of H9c2 cells stimulated with LPS (20 *μ*g/mL, 24 h) was observed in the presence and absence of the estrogen receptor-specific antagonist ICI182,780. The results show that medium to high doses of adenosine can significantly promote cardiac function (LVEF and LVFS) and reduce the levels of inflammatory factors (TNF-*α*, IL-6, PCT, and cTnI) and p-JNK in septic rats, with a significant difference seen between male and female rats. The results of flow cytometry show that adenosine significantly inhibited increases in ROS levels, mitochondrial membrane potential, and the swelling degree of mitochondria in H9c2 cells stimulated with LPS, but this effect could be blocked by ICI182,780, indicating that adenosine attenuated LPS-induced cardiac dysfunction by inhibiting mitochondrial function via the ER pathway.

## 1. Introduction

Sepsis is a life-threatening organ dysfunction syndrome caused by an abnormal immune response to infection [1] and is one of the major causes of death in critically ill patients. According to the latest statistical data [2], the mortality rate of patients with sepsis is as high as 24.3%, and the occurrence of sepsis-induced cardiomyopathy is the primary cause of death. In a study by Pulido et al. [3], the recorded incidence of sepsis-induced cardiomyopathy was reported to be as high as 64%. In recent years, with the development of technology monitoring cardiac function, a greater understanding of sepsis-induced cardiomyopathy has been reached. The heart is an organ rich in mitochondria, and the mitochondrial dysfunction in sepsis is therefore receiving increasing attention [4]. Studies have shown that many factors are associated with the development of myocardial damage in sepsis, such as excessive production of ROS and destruction of mitochondrial membrane potential [5, 6]; however, the specific underlying mechanism of myocardial damage in patients with sepsis remains unclear. Gram-negative bacteria which could secrete lipopolysaccharide are the main pathogens that induce sepsis. In this study, LPS was used to induce sepsis.

Adenosine, a compound produced by linking the N-9 of adenine to the C-1 of D-ribose with a *β*-glycosidic bond, is an endogenous nucleoside distributed throughout human cells and can be directly phosphorylated in the myocardium, forming adenylate [7]. Adenosine has a physiological effect in many systems (e.g., cardiovascular system) and tissues. For instance, adenosine slows atrioventricular node conduction, blocks atrioventricular node reentry, and restores the normal sinus rhythm of patients with paroxysmal supraventricular tachycardia (PSVT) (with or without preexcitation syndrome) [8, 9]. Whether adenosine can contribute to protection of cardiomyopathy induced by LPS has not been reported. In our previous studies, it has been reported that adenosine has estrogen-like effects mediated by the estrogen receptor (ER) [10]. Other studies have shown that drugs with estrogen-like activity can protect the immune system of mice with traumatic blood loss and reduce the occurrence of immunosuppression [11, 12]. Furthermore, estradiol (E2) has been demonstrated to modulate the function of LPS-treated macrophages and H9c2 cells [13, 14], suggesting a possible link between estrogen-like activity and sepsis. Nevertheless, it remains to be clarified whether the estrogen-like effects of adenosine can contribute to protection of cardiac dysfunction associated with LPS-induced sepsis. This study was designed to examine the effects of adenosine on LPS-induced cardiac anomalies and the underlying mechanisms involved.

Astragali Radix (named Huang Qi in China, called HQ in this study) is a traditional Chinese medicine that has the effect of supplementing qi, mainly containing astragalus saponins, sucrose, astragalus polysaccharides, amino acids, selenium, zinc, copper, and so on [15]. Researches have reported that HQ have many kinds of pharmacological activities, such as strong heart, diuresis, and antithrombotic, fight free radical damage, protect myocardium, and enhance the immune function [16]. HQ is used for treatment of myocardial injury, such as sepsis, diabetes, and hypertension [17]. In addition, HQ has estrogen-like activity. In our study, HQ is a positive control drug.

## 2. Materials and Methods

### 2.1. Plant Material and Reagents

Adenosine and LPS were purchased from Sigma (St Louis, MO, USA); Huangqi Zhusheye (referred to HQ, positive control group in this study) was purchased from Zbd Pharmaceutical Co., Ltd. (HeiLongJiang, China). Dulbecco's modified Eagle medium (DMEM, Gibco, Pittsburgh, PA, USA), heat-inactivated fetal calf serum (HyClone, Logan, UT, USA), methyl thiazolyl tetrazolium (MTT) and dimethyl sulfoxide (DMSO) (Amresco, Seattle, WA, USA), ICI182,780 (Tocris, Bristol, UK), and Salirasib (FST, APExBIO, Amazon, USA). A microplate reader was also used (Bio-Rad, Hercules, CA, USA) were used.

### 2.2. Cell Culture and Treatment

H9c2 cells for* in vitro* experiments were purchased from ATCC (Rockville, MD, USA) and cultured in DMEM supplemented with 50 units/mL penicillin, 50 *μ*g/mL streptomycin and 10% heat-inactivated fetal calf serum at 37°C in a humidified incubator with 5% CO_2_ (Thermo Scientific, Waltham, MA, USA). There were four experimental groups in the present study: control, model (LPS 20 *μ*g/mL), HQ (0.1 mg/mL, LPS 20 *μ*g/mL, positive control), and adenosine (10*μ*M, LPS 20 *μ*g/mL) groups. H9c2 cells were seeded on a 100 mm × 20 mm cell culture flask at a density of 2×10^5^ cells/mL. Following treatment with HQ and adenosine for 24 h, the cells were collected. For antagonism, the estrogen antagonist Faslodex (ICI182,780, 1 *μ*M; ER nonspecific) was added 30 min prior to treatment with HQ and adenosine to evaluate whether the observed effects were mediated by the ER. Cells from each group were used for mitochondrial swelling, membrane potential (ΔΨm), and ROS detection assays.

### 2.3. Evaluation of the Degree of Mitochondrial Swelling [18]

To determine the large amplitude swelling of H9c2 cells, the isolation of the mitochondria and the cytosol was performed using a Cell Mitochondria Isolation kit (SM0020; Beijing Solarbio Science & Technology Co., Ltd., China). Briefly, H9c2 cells in each group were incubated in ice-cold mitochondrial lysis buffer for 20 min. The cell suspension was transferred to a glass homogenizer and homogenized for 20 strokes. The homogenate was subjected to centrifugation at 800 x g for 15 min at 4°C to remove the nuclei and unbroken cells. The supernatant was collected and centrifuged again at 12,000 x g for 15 min at 4°C to obtain the mitochondrial fraction. Samples of mitochondria were dissolved in buffer and subjected to flow cytometry (FCM; BD FACS Aria III; BD Biosciences, USA).

### 2.4. Evaluation of Mitochondrial Membrane Potential (ΔΨm) [18]

ΔΨm was assessed using FCM with 5,5′,6,6′-tetrachloro-1,1′,3,3′-tetraethylbenzimidazole-carbocyanide iodine staining (JC-1; GK3610; Genview; Beijing, China). H9c2 cells in each group were stained with JC-1 for 20 min at 37°C and subjected to FCM. JC-1 (red fluorescence) represented normal cells, and monomeric JC-1 (green fluorescence) represented cells in which ΔΨm was increased.

### 2.5. Determination of Intracellular ROS Levels

The production of ROS in H9C2 cells was fluorometrically monitored using the nonfluorescent probe, 2′,7′-dichlorofluorescein diacetate (DCFH-DA) (CA1410; Beijing Solarbio Science & Technology Co., Ltd, China). DCFH-DA passively diffuses into cells and is deacetylated, becoming a fluorescent compound, 2′,7′-dichlorofluorescein (DCFH). DCFH reacts with ROS to form the fluorescent product, DCF, which is trapped inside the cells. Cells in each group cultured on 6-well dishes were trypsinized and collected by centrifugation. DCFH-DA, diluted in DMEM to a final concentration of 1 *μ*M, was added to the H9c2 cells and incubated at 37°C for 20 min. Subsequently, H9c2 cells were washed three times with PBS, and DCF fluorescence was measured by FCM. Mean fluorescence intensity values represented the levels of intracellular ROS.

### 2.6. Animals

The present study was conducted in accordance with the Regulations of Experimental Animal Administration issued by the State Committee of Science and Technology of the People's Republic of China. Male (6 weeks old; 200 ± 20 g) and female (6 weeks old; 180 ± 20 g) Wistar rats were obtained from Beijing Vital River Laboratory Animal Technology Co., Ltd. (Ethical approval reference number: SCXK2016-0011). All rats were housed in cages on a 12 h/12 h light-dark schedule at a controlled temperature (22°C) with free access to food and water at the Laboratory Animal Research Center of Henan University of Chinese Medicine. All the procedures for the care of the rats were in accordance with the institutional guidelines for animal use in research. Following adaptive feeding for one week, 75 male and 75 female rats received LPS (10 mg/kg, i.p.) induced the sepsis disease, and were divided into five groups, with 15 female and 15 male rats in each: model (LPS 10 mg/kg, i.p), HQ (2000 mg/kg + LPS 10 mg/kg, i.p.), low-dose adenosine (Ad, 25 mg/kg + LPS 10 mg/kg, i.p), mid-dose adenosine (Ad, 50 mg/kg+ LPS 10 mg/kg, i.p), and high-dose adenosine (Ad, 100 mg/kg+ LPS 10 mg/kg, i.p) groups. In addition, 10 female and 10 male rats received the same volume of saline (i.p.) as a control group. Two hours later, treatment rats were gavaged with different doses of adenosine, while control rats were gavaged with water. The doses were administered as described above twice a day for 3 consecutive days. Eighteen hours after the final administration, the animals were anesthetized with ketamine hydrochloride to measure heart function, and blood was subsequently collected using the abdominal aortic method. The carefully dissected hearts were weighed on a sensitive torsion balance and immediately frozen in liquid nitrogen at -80°C.

### 2.7. Cytometric Bead Array (CBA)

CBA was performed according to the manufacturer's instructions. Heart tissue was incubated with lysate treatment reagent (6299995; BD Biosciences; New York, USA) on ice for 15 minutes to release the intracellular phosphorylated extracellular-regulated protein kinases 1/2 (p-ERK1/2), c-Jun N-terminal kinase (p-JNK), and p-p38 proteins into the supernatant. A total of 50 *μ*L heart tissue lysate from each sample was incubated with 50 *μ*L anti-p-ERK1/2 (7205548; BD), anti-p-JNK (7170701; BD), or anti-p-p38 (7202572; BD) antibody conjugated to beads and 50 *μ*L PE-labeled anti-p-ERK1/2, anti-p-JNK, and anti-p-p38 in the dark at room temperature for 2 hours with sufficient shaking. Following washing with CBA wash buffer, the samples were resuspended in 500 *μ*L CBA wash buffer and analyzed immediately by FCM using the Cell Quest software (BD). The Tracking Beads system was used according to the manufacturer's guidelines.

### 2.8. ELISA

Plasma was collected and used to detect the levels of interleukin 6 (IL-6; R180109-003a; Neobioscience Co., Ltd., Shenzhen, China), tumor necrosis factor *α* (TNF-*α*; R180109-102a; Neobioscience Co., Ltd., Shenzhen, China), procalcitonin (PCT; CSB-E13419r; CUSABIO BIOTECH CO., Ltd., Wuhan, China), and cardiac troponin I (cTnI; 2HSPA1018; Life Diagnostics, lnc., West Chester, PA) according to the respective manufacturer's instructions.

### 2.9. Statistical Analysis

Analyses were performed using the SPSS 20.0 software (IBM; New York, NY, USA). Statistical significance was assessed in comparison with the respective control for each experiment using one-way ANOVA. A* p* value less than 0.05 was accepted as significant.

## 3. Results

### 3.1. Effect of Adenosine on Heart Function in LPS-Induced Septic Rats

**Figure 1:**
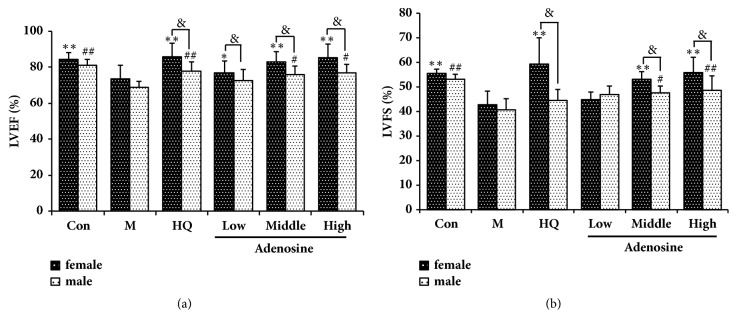
Effects of adenosine on heart function in LPS-induced septic rats as assessed by ultrasound. Female and male rats received LPS (10 mg/kg, i.p., 2 h) prior to gavage with different doses of adenosine. (a) Effects of different doses of adenosine on LVEF. (b) Effects of different doses of adenosine on LVFS. X ± SD, n = 8 rats per group, ^*∗*^*P *< 0.05, ^*∗∗*^*P *< 0.01 versus female model group; ^#^*P *< 0.01, ^##^*P *< 0.01 versus male model group; ^&^*P *< 0.05 versus male rats in each group.

Ultrasound revealed that LPS challenge significantly decreased left ventricular ejection fraction (LVEF) and left ventricular fractional shortening (LVFS), the effect of which was abrogated by adenosine. As a positive control, HQ also increased LVEF and LVFS in LPS-induced septic rats. Interestingly, the effects of HQ and adenosine were different between male and female rats; HQ and adenosine displayed a better effect on heart function in female rats, as shown in Figure 1.

### 3.2. Effects of Adenosine on Proinflammatory Cytokines in LPS-Induced Septic Rats

**Figure 2:**
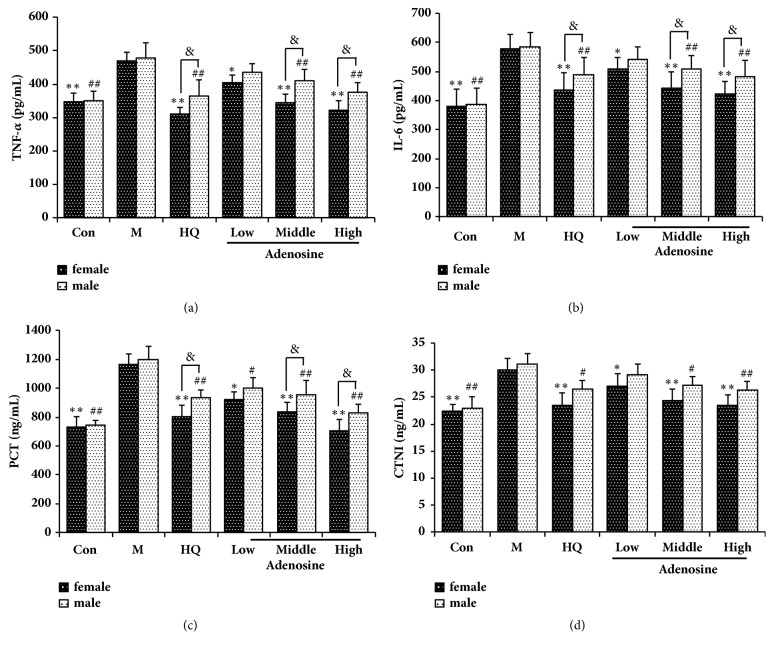
Effects of adenosine on proinflammatory cytokine levels in LPS-induced septic rats as assessed by ELISA. Female and male rats received LPS (10 mg/kg, i.p., 2 h) prior to gavage with different doses of adenosine. (a) Effects of different doses of adenosine on TNF-*α*. (b) Effects of different doses of adenosine on IL-6. (c) Effects of different doses of adenosine on PCT. (d) Effects of different doses of adenosine on cTnI. X ± SD, n = 8 rats per group, ^*∗*^*P *< 0.05, ^*∗∗*^*P *< 0.01 versus female model group; ^#^*P *< 0.01, ^##^*P *< 0.01 versus male model group; ^&^*P *< 0.05 versus male rats in each group.

To assess the effect of adenosine on proinflammatory cytokine production, the levels of TNF*α*, IL-6, PCT, and cTnI were evaluated by ELISA. As shown in Figure 2, LPS significantly elevated the levels of all the measured proinflammatory cytokines, while HQ and adenosine attenuated this increase, thereby conferring cardioprotection. Moreover, we also observed that the effect of HQ and adenosine showed better effects of mitigated cytokines in female rats.

### 3.3. Effects of Adenosine on the MAPK Signaling Pathway in LPS-Induced Septic Rats

**Figure 3:**
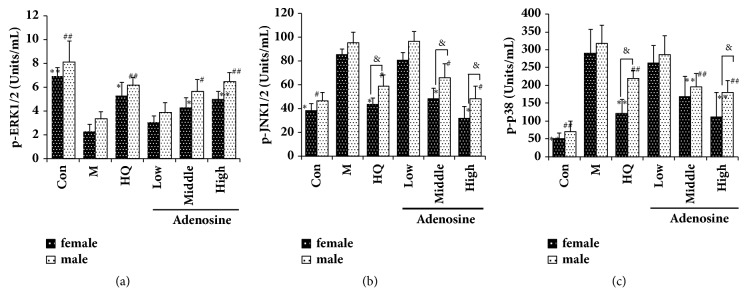
Effects of adenosine on the MAPK signaling pathway in LPS-induced septic rats as assessed by CBA. Female and male rats received LPS (10 mg/kg, i.p., 2 h) prior to gavage with different doses of adenosine. (a) Effects of different doses of adenosine on the phosphorylation of ERK. (b) Effects of different doses of adenosine on the phosphorylation of JNK. (c) Effects of different doses of adenosine on the phosphorylation of p38. X ± SD, n = 8 rats per group, ^*∗*^*P *< 0.05, ^*∗∗*^*P *< 0.01 versus female model group; ^#^*P *< 0.01, ^##^*P *< 0.01 versus male model group; ^&^*P *< 0.05 versus male rats in each group.

To investigate whether the MAPK signaling pathway contributes to the cardioprotection offered by adenosine against LPS treatment, the levels of phosphorylated stress signaling molecules p38, ERK, and JNK were evaluated by CBA using flow cytometry. The results demonstrated that HQ and adenosine decreased the phosphorylation of JNK and p38 and increased the phosphorylation of ERK. In addition, the effects of HQ and adenosine on the phosphorylation levels of all three kinases were different between male and female rats, the latter of which had a better prognosis. The results are shown in Figure 3.

### 3.4. The Antagonistic Activity of ICI182,780 against Adenosine on the Mitochondrial Swelling in LPS-Stimulated H9c2 Cells

**Figure 4:**
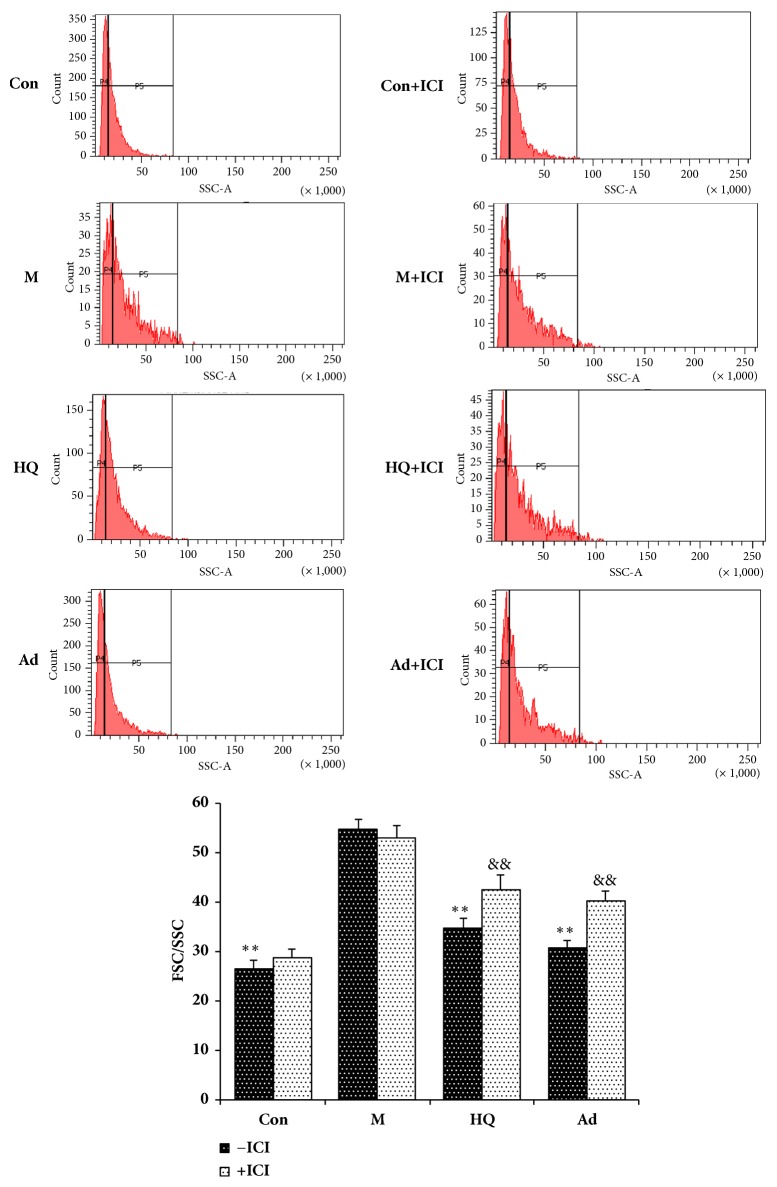
The antagonistic activity of ICI182,780 against adenosine on the mitochondrial swelling in LPS-stimulated H9c2 cells as assessed by FCM. H9c2 cells seeded on a 100 mm × 20 mm cell culture flask at a density of 2×10^5^ cells/mL were treated with HQ (0.1 mg/mL) and adenosine (10 *μ*M) for 24 h. In another experiment, the ER nonspecific antagonist Faslodex (ICI182,780, 1 *μ*M) was added 30 min prior to HQ (0.1 mg/mL) and adenosine (10 *μ*M) to evaluate whether the observed effects elicited by HQ and adenosine were mediated by the ER. Cells in each group were collected 24 h later and mitochondrial swelling was assessed. X ± SD, n = 3, ^*∗∗*^*P *< 0.01 versus model group (-ICI); ^&&^*P *< 0.05 versus each group.

With the aim of understanding mitochondrial function more objectively, mitochondrial swelling was evaluated by FCM the following determination of the FSC-SSC parameters. FSC correlates highly with cell size or volume, and SSC is related to granularity and the refractive index. The quantitative degree of mitochondrial swelling was represented by the FSC/SSC ratio. LPS significantly elevated the levels of mitochondrial swelling in H9c2 cells, while HQ and adenosine attenuated this increase. Moreover, ICI182,780 could block the effect of HQ and adenosine on mitochondrial swelling but had no effect alone. The results are shown in Figure 4.

### 3.5. The Antagonistic Activity of ICI182,780 against Adenosine on the MMP (ΔΨm) in LPS-Stimulated H9c2 Cells

**Figure 5:**
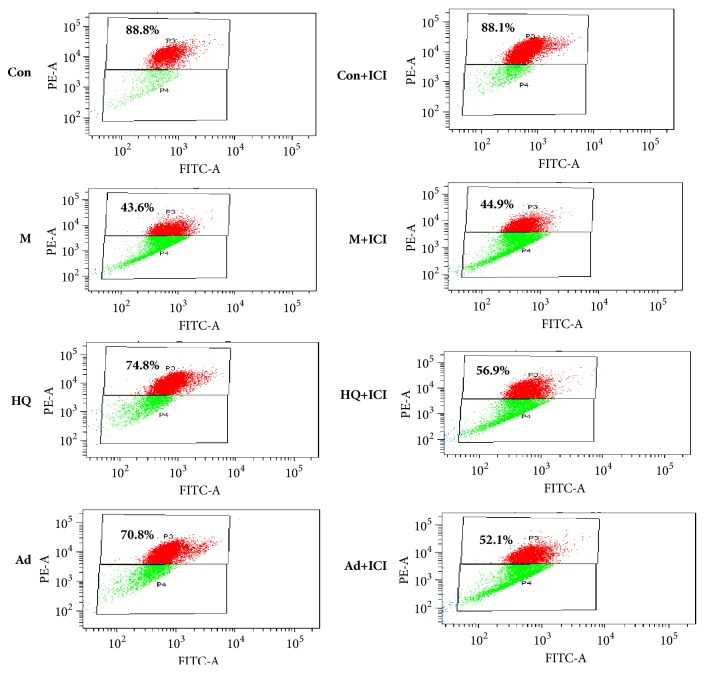
The antagonistic activity of ICI182,780 against adenosine on MMP (or ΔΨm) in LPS-stimulated H9c2 cells as assessed by FCM. H9c2 cells seeded on a 100 mm × 20 mm cell culture flask at a density of 2×10^5^ cells/mL were treated with HQ (0.1 mg/mL) and adenosine (10 *μ*M) for 24 h. In another experiment, the ER nonspecific antagonist Faslodex (ICI182,780, 1 *μ*M) was added 30 min prior to HQ (0.1 mg/mL) and adenosine (10 *μ*M) to evaluate whether the observed effects elicited by HQ and adenosine were mediated by the ER. Cells in each group were collected 24 h later and ΔΨm was assessed.

For a more objective understanding of MMP, FCM was used to detect ΔΨm; JC-1 aggregates in the mitochondria and emits red fluorescence, which can be easily monitored. Treatment of H9c2 cells with LPS for 24 h resulted in the dissipation of MMP, as shown by reduced JC-1 staining. The red fluorescence intensity in the HQ and adenosine groups was stronger than that in the LPS group, indicating that LPS significantly elevated the levels of ΔΨm in H9c2 cells, while HQ and adenosine attenuated this increase. In addition, ICI182,780 could block the effect of HQ and adenosine on ΔΨm, but had no effect alone. The results are shown in Figure 5.

### 3.6. The Antagonistic Activity of ICI182,780 against Adenosine on ROS Production in LPS-Stimulated H9c2 Cells

**Figure 6:**
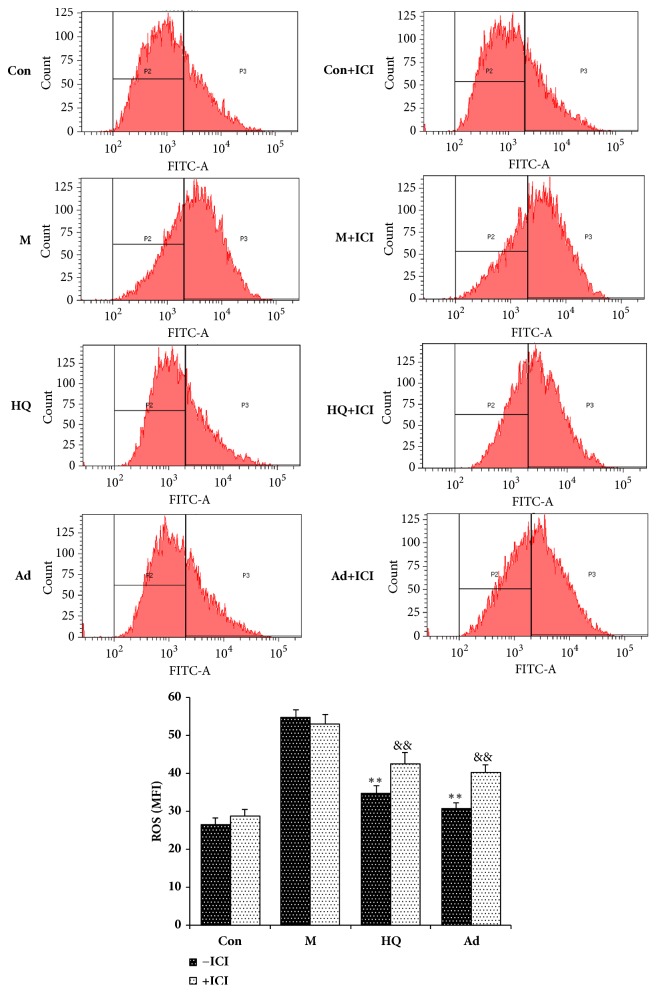
The antagonistic activity of ICI182,780 against adenosine on ROS production in LPS-stimulated H9c2 cells as assessed by FCM. H9c2 cells seeded on a 100 mm × 20 mm cell culture flask at a density of 2×10^5^ cells/mL were treated with HQ (0.1 mg/mL) and adenosine (10 *μ*M) for 24 h. In another experiment, the ER nonspecific antagonist Faslodex (ICI182,780, 1 *μ*M) was added 30 min prior to HQ (0.1 mg/mL) and adenosine (10 *μ*M) to evaluate whether the observed effects elicited by HQ and adenosine were mediated by the ER. Cells in each group were collected 24 h later and ROS levels were assessed. X ± SD, n = 3, ^*∗∗*^*P *< 0.01 versus model group (-ICI); ^&&^*P *< 0.05 versus each group.

To explore the molecular mechanism of adenosine, the levels of ROS were detected by FCM. As shown in Figure 6, LPS significantly elevated the levels of ROS in H9c2 cells, while HQ and adenosine attenuated this increase. Moreover, ICI182,780 could block the effects of HQ and adenosine on ROS production to differing degrees, but had no effect alone.

## 4. Discussion

Sepsis-induced myocardial dysfunction (SIMD) is an important predictive factor for the prognosis of sepsis [19]. SIMD is a common complication of severe sepsis, with clinical manifestations including myocardial damage and cardiac dysfunction. The mechanism of SIMD is very complicated; excessive release of cytokines [20], excessive activation of complement [21], apoptosis of myocardial cells [22], and an imbalance in energy metabolism are all considered related factors [23]. Through clinical research, Edo Y et al. observed a reduction in LVEF and left ventricular dilatation in patients in the early stage of sepsis [24]. Due to the portability and noninvasiveness of the echocardiograph, it has become the most commonly used tool for the assessment of cardiac function. Therefore, in the present study, the effect of adenosine on cardiac function in septic rats was first assessed by echocardiography. The results show that HQ (2000 mg/kg) and adenosine (50 and 100 mg/kg) can significantly improve LVEF and LVFS in septic rats, thus promoting their cardiac function. Interestingly, the therapeutic effect of HQ and adenosine in female septic rats was significantly superior to that in male septic rats (*P* < 0.05).

Gram-negative bacteria [25] are the main pathogens that induce sepsis. The lipopolysaccharides (LPS) located in the cell wall of these bacteria are lipid and polysaccharide complexes that can activate mononuclear cells and macrophages, causing many pathophysiological changes, including the release of a large number of proinflammatory factors such as TNF-*α* and IL-6. Procalcitonin (PCT) is a nonhormone glycoprotein that is released into the circulatory system of patients with severe systemic infections, particularly those caused by bacteria, and is used as the most accurate serological diagnostic marker for sepsis to judge the type and severity of infection [26]. cTnI is a marker of myocardial injury, with high sensitivity and specificity, and is of great significance for the diagnosis and risk stratification of myocardial infarction. In recent years, studies have found that cTnI is elevated in patients with sepsis-related cardiomyopathy, and its increase often reflects the severity of myocardial cell injury, which is closely related to disease severity and mortality [27]. The results show that HQ (2000 mg/kg) and adenosine (50 and 100 mg/kg) significantly reduced serum TNF-*α*, IL-6, PCT, and cTnI levels in septic rats. Similarly, HQ and adenosine had a significant male−female difference in the regulation of serum inflammatory factors in septic rats (*P* < 0.05).

Mitogen-activated protein kinases (MAPKs) are serine/threonine-specific and are widely distributed in eukaryotic cells. Typical MAPKs include c-Jun-N-terminal kinase (JNK), p38 kinases, and extracellular signal transduction kinase (ERK). Serine/threonine-specific protein kinases can be activated by extracellular signals or stimuli such as physical stress, proinflammatory cytokines, growth factors, and bacterial complexes, and this signal is subsequently amplified to the nucleus by a phosphorylation cascade. Therefore, MAPKs indirectly regulate the activity of transcription factors, resulting in altered expression of corresponding genes and regulation of various important cell physiological/pathological processes such as cell growth, cell differentiation, environmental stress adaptation, and inflammatory response [28, 29]. The results of the present study show that HQ (2000 mg/kg) and adenosine (50 and 100 mg/kg) could significantly reduce the levels of p-JNK and p-P38 and increase the level of p-ERK in septic rats. Moreover, the effect of adenosine on p-JNK levels also showed a significant male−female difference (*P* < 0.05). It can be speculated that adenosine may play a protective role in septic rats through the JNK pathway.

Gender differences can cause changes and differences in the pharmacokinetics and pharmacodynamics of endogenous and exogenous compounds. Female animals contain higher levels of estrogen, which affects the bioavailability, distribution, absorption, metabolism, and excretion of drugs. In order to rule out this interference, we observed the effects of adenosine on male and female rats induced by LPS, respectively. Interestingly, results showed that the therapeutic effect of adenosine in female septic rats was significantly superior to that in male septic rats. This might be associated with higher levels of estrogen receptors in female mice; adenosine also has estrogen-like activity [10]. Subsequently, the underlying mechanisms of adenosine on the mitochondrial function of H9c2 cells stimulated with LPS were observed in the presence and absence of the estrogen receptor-specific antagonist ICI182,780.

In recent years, mitochondrial dysfunction has been recognized as one of the major factors in the pathogenesis of sepsis. The reported mechanism includes excessive ROS production during sepsis, which causes oxidative stress injury and results in oxidative damage to the mitochondrial respiratory chain and insufficient ATP production, thereby impairing mitochondrial membrane potential and ultimately leading to mitochondrial permeability transition, mitochondrial energetic dysfunction, and organelle swelling. Our FCM results show that HQ (0.1mg/mL) and adenosine (10 *μ*M) could significantly reduce ROS levels and mitochondrial membrane potential and inhibit the increase in mitochondrial swelling in H9c2 cells induced by LPS (20 *μ*g/mL, 24 h). This effect could be blocked by the specific estrogen receptor antagonist ICI182,780, suggesting that the protective effect of adenosine in H9c2 cells is mediated by the ER.

Previous studies have shown that the condition of sepsis is closely related to gender. In comparison with male septic animals, the prognosis is better for females, which may be explained by the fact that female animals have more anti-inflammatory factors, such as interleukin 10 (IL-10), and drugs with estrogen-like activity can protect the immune system of mice with blood loss, reducing the occurrence of immunosuppression. This indicates that there may be a connection between estrogen-like activity and sepsis symptoms. Accordingly, previous studies in our laboratory have shown that adenosine has estrogen-like activity and that its function is mediated by estrogen receptors. The present study demonstrates that there was a male−female difference in the therapeutic effect of HQ and adenosine in septic rats and that the improvement in mitochondrial function in H9c2 cells elicited by HQ and adenosine could be antagonized by ICI182,780. It is reasonable to suggest, therefore, that HQ and adenosine executes its protective function via the ER pathway.

In our study, HQ is a positive control drug. HQ has estrogen-like activity which was used for treatment of myocardial injury, such as sepsis, diabetes, and hypertension. Results of animal experiments and cell experiments also showed that HQ attenuated LPS-induced cardiac dysfunction by inhibiting mitochondrial function via the ER pathway. Moreover adenosine has the same molecular mechanism as HQ.

## 5. Conclusion

Medium and high doses of adenosine could significantly promote cardiac function (LVEF and LVFS) and reduce inflammatory factors (TNF-*α*, IL-6, PCT, and cTnI) and p-JNK levels in septic rats, with an obvious male−female difference. The results of experiments using ICI182,780 to antagonize the effect of adenosine on LPS-stimulated H9c2 cells show that adenosine could significantly inhibit the increases in ROS levels, mitochondrial membrane potential, and mitochondrial swelling and that this effect could be blocked by the estrogen receptor-specific antagonist ICI182,780. In conclusion, adenosine attenuated LPS-induced cardiac dysfunction by inhibiting mitochondrial function via the ER pathway.

## Data Availability

The data used to support the findings of this study are available from the corresponding author upon request.
